# Clarification and Complement to “Mean-Field Description and Propagation of Chaos in Networks of Hodgkin–Huxley and FitzHugh–Nagumo Neurons”

**DOI:** 10.1186/s13408-015-0031-8

**Published:** 2015-09-01

**Authors:** Mireille Bossy, Olivier Faugeras, Denis Talay

**Affiliations:** TOSCA Laboratory, INRIA Sophia Antipolis – Méditerranée, Route des lucioles, Sophia Antipolis, France; NeuroMathComp Laboratory, INRIA Sophia Antipolis – Méditerranée, Route des lucioles, Sophia Antipolis, France

**Keywords:** Mean-field limits, Propagation of chaos, Stochastic differential equations, Neural networks, Neural assemblies, Hodgkin–Huxley neurons, FitzHugh–Nagumo neurons

## Abstract

In this note, we clarify the well-posedness of the limit equations to the mean-field *N*-neuron models proposed in (Baladron et al. in J. Math. Neurosci. 2:10, 2012) and we prove the associated propagation of chaos property. We also complete the modeling issue in (Baladron et al. in J. Math. Neurosci. 2:10, 2012) by discussing the well-posedness of the stochastic differential equations which govern the behavior of the ion channels and the amount of available neurotransmitters.

## Introduction

The paper of Baladron et al. [1] studies quite general networks of neurons and aims to prove that these networks propagate chaos in the sense originally developed by Sznitman [2] after the seminal work of Kac on mean-field limits and McKean’s work [3] on diffusion processes propagating chaos. As observed by the authors, the membrane potentials of the neurons in the networks they consider are described by interacting stochastic particle dynamics. The coefficients of these McKean–Vlasov systems are not globally Lipschitz. Therefore the classical results of the propagation of chaos theory do not directly apply and a specific analysis needs to be performed. The main theorems (existence and uniqueness of the limit system when the number of particles tends to infinity, propagation of chaos property) are stated under a fairly general hypothesis on the coefficients. Unfortunately the proof in [1], pp. 24–25, involves an erroneous management of hitting times in combination with a truncation technique, and the limit system may not be well defined under the too general hypothesis used by the authors. Indeed, the following equation, where *ϕ* is a bounded and locally Lipschitz function and $Z_{0}$ is a random variable, satisfies the hypothesis made in [1], pp. 15–16:

$$Z_{t} = Z_{0} + \int_{0}^{t} \mathbb {E}\phi(Z_{s})\,ds. $$

However, Scheutzow exhibited examples of a function *ϕ* and initial condition $Z_{0}$ for which many solutions do exist: see Counterexample 2 in [4] and the remark which follows it.^1^

This note restricts the neuron model to the much used variants of the FitzHugh–Nagumo and Hodgkin–Huxley models. Our objective is two-fold: first, we discuss a modeling issue on the diffusion coefficients of the equations describing the proportions of open and closed channels that guarantees that these variables do not escape from the interval $[0,1]$. This was not completely achieved in [1] and can be seen as a complement to this paper.

Second, we give a rigorous proof of the propagation of the chaos property.

## The Models

In this section we present and discuss the stochastic models considered by Baladron et al. [1] for the electrical activity of *p̅* populations of neurons. Each population has a label *α* and $N_{\alpha}$ elements. We denote by $\mathcal {P}$ the set of the *p̅* population labels and by $N:=\sum_{\alpha\in \mathcal {P}} N_{\alpha}$ the total number of neurons.

Given the neuron *i* in a population *α*, the stochastic time evolution of the membrane potential is denoted by $(V^{i}_{t})$. In the case of the Hodgkin–Huxley model, the sodium and potassium activation variables, which represent proportions of open gates along the neuron *i* are, respectively, denoted by $(n^{i}_{t})$, $(m^{i}_{t})$. The sodium inactivation variable, which is also a proportion of open gates, is denoted by $(h^{i}_{t})$. In the case of the FitzHugh–Nagumo model, the recovery variable is denoted by $(w^{i}_{t})$. Both models feature synaptic variables $(y^{i}_{t})$ which represent the proportion of available neurotransmitters at the synapses of neuron *i*.

The synaptic connections between neurons are assumed to be chemical in [1]. We make the same assumption here. This implies that the synaptic current $I_{ij}^{\text{syn}}$ from the presynaptic neuron *j* in population *γ* to the postsynaptic neuron *i* in population *α* can be written

$$I_{ij}^{\text{syn}}(t)=-g_{ij}(t) \bigl(V^{i}_{t}- \overline{V}^{\alpha\gamma}\bigr), $$

where $\overline{V}^{\alpha\gamma}$ is the synaptic reversal potential of the $j \to i$ synapse, assumed to be approximately constant across populations, and $g_{ij}(t)$ the electric conductance of that synapse. Hence the postsynaptic neuron *i* belongs to population *α* and the presynaptic neuron *j* to population *γ*. This conductance is the product of the maximal conductance, noted $J^{ij}_{t}$, of the synapse by the proportion $y^{j}_{t}$ of neurotransmitters available at neuron *j*. Conductances are positive quantities.

The processes $(V^{i}_{t},n^{i}_{t},m^{i}_{t},h^{i}_{t},w^{i}_{t},y^{i}_{t})$ are defined by means of the stochastic differential systems (1) or (4) in the *N*-neuron model section below. The mean-field limit processes are defined in (9) and (5). Well-posedness of those systems is postponed to Sect. 4.

### The *N*-Neuron Model

The variants of the FitzHugh–Nagumo and Hodgkin–Huxley dynamics proposed in Baladron et al. [1] to model neuron networks are all of the two types below; the only differences concern the algebraic expressions of the function $F_{\alpha}$ and the fact that the FitzHugh–Nagumo model does not depend on the variables $(n_{t}^{i},m_{t}^{i},h_{t}^{i})$ but on the recovery variable $(w^{i}_{t})$ only. Conversely, the Hodgkin–Huxley model does not depend on $w^{i}_{t}$. In what follows we denote by **q** the vector $(n,m,h)$ of $\mathbb {R}^{3}$ in the case of the Hodgkin–Huxley model, and the real $(w)$ in the case of the FitzHugh–Nagumo model. We also note $(\mathbf {W}^{i}_{t}) = (W^{i,V}_{t},W^{i,y}_{t},W^{i,n}_{t},W^{i,m}_{t},W^{i,h}_{t})$, independent Brownian motions ($1\leq i\leq N$).

Given a neuron *i*, the number $p(i)=\alpha$ denotes the type of the population it belongs to.

Equations (1) and (4) below are those studied in [1]. They correspond to two different models for the maximum conductances. The first one does not respect the positivity constraint while the second one guarantees that these quantities stay positive. In these equations, all quantities which are not time indexed are constant parameters.

#### Simple Maximum Conductance Variation

For *i* and *j* such that $p(i) = \alpha$ and $p(j)=\gamma$, the model assumes that $J^{ij}_{t}$ fluctuates around a mean $\overline {J}^{\alpha\gamma}$ according to a white noise with standard deviation $\sigma^{J}_{\alpha\gamma}$:

$$J^{ij}_{t}=\overline{J}^{\alpha\gamma}+\sigma^{J}_{\alpha\gamma} \frac {dB^{i \gamma}_{t}}{dt}. $$

For $(B^{i\gamma}_{t})$ a family of independent Brownian motions, independent of the Brownian family $(\mathbf {W}^{i}_{t})$, the equations describing the dynamics of the state vector of neuron *i* in population *α* are

1 $$ \textstyle\begin{cases} \mbox{for $i$ such that } p(i) = \alpha, \mbox{ for }\mathbf {q}= (w) \mbox{ or } (n,m,h), \\ dV^{i}_{t} = F_{\alpha}(t, V^{i}_{t} , \mathbf {q}^{i}_{t})\,dt - \sum_{ \gamma\in \mathcal {P}} (V^{i}_{t} - \overline{V}^{\alpha\gamma}) \frac{\overline{J}^{\alpha\gamma}}{N_{\gamma}} (\sum_{j=1}^{N} {\mathbb {1}}_{\{p(j)=\gamma\}}y^{j}_{t} )\,dt \\ \hphantom{dV^{i}_{t} =}{}- \sum_{ \gamma\in \mathcal {P}} (V^{i}_{t} - \overline{V}^{\alpha\gamma}) \frac{\sigma^{J}_{\alpha\gamma}}{N_{\gamma}} ( \sum_{j=1}^{N} {\mathbb {1}}_{\{p(j) = \gamma\}}y^{j}_{t} )\,dB^{\gamma,i}_{t} + \sigma_{\alpha}\,dW^{i,V}_{t}, \\ dy^{i}_{t} = (a^{\alpha}_{r}S_{\alpha}(V^{i}_{t}) (1-y^{i}_{t}) - a^{\alpha}_{d}y^{i}_{t} )\,dt \\ \hphantom{dy^{i}_{t} =}{}+ \sqrt{ \vert a^{\alpha}_{r} S_{\alpha}(V^{i}_{t}) (1-y^{i}_{t}) + a^{\alpha}_{d}y^{i}_{t} \vert } \chi(y^{i}_{t})\,dW^{i,y}_{t}, \end{cases} $$

coupled with

2 $$ dw^{i}_{t} = c_{\alpha}\bigl(V^{i}_{t} + a_{\alpha}- b_{\alpha}w^{i}_{t}\bigr)\,dt $$

or

3 $$\begin{aligned} dx^{i}_{t} ={}& \bigl( \rho_{x}\bigl(V^{i}_{t}\bigr) \bigl(1-x^{i}_{t}\bigr) - \zeta_{x} \bigl(V^{i}_{t}\bigr)x^{i}_{t} \bigr) \,dt \\ &{}+ \sqrt{\bigl\vert \rho_{x}\bigl(V^{i}_{t} \bigr) \bigl(1-x^{i}_{t}\bigr) + \zeta_{x} \bigl(V^{i}_{t} \bigr) x^{i}_{t}\bigr\vert } \chi\bigl(x^{i}_{t}\bigr)\,dW^{i,x}_{t}\quad \mbox{ for } x=n,m,h. \end{aligned}$$

The reader may wonder about the reason for the square root term and the function *χ* in the diffusion coefficient of the SDE for the processes $x^{i}$ and $y^{i}$. The square root arises from the fact that this SDE is a Langevin approximation to a stochastic hybrid, or piecewise deterministic, model of the ion channels. There is a finite (albeit large) number of such ion channels and each of them can be modeled as jump Markov processes coupled to the membrane potential. Altogether ion channels and membrane potentials are described by a piecewise deterministic Markov process which, as shown for example in [6], can be approximated by the solution to the SDE shown in (1) and (4). Hypothesis 2.1(i) below on the function *χ* implies that the processes $x^{i}$ and $y^{i}$ are valued in $[0,1]$: see Sect. 4.

#### Sign-Preserving Maximum Conductance Variation

4 $$ \textstyle\begin{cases} \mbox{For }i\mbox{ such that }p(i)=\alpha\mbox{,}\mbox{ for }\mathbf {q}= (w) \mbox{ or } (n,m,h), \\ dV^{i}_{t} = F_{\alpha}(t, V^{i}_{t} , \mathbf {q}^{i}_{t})\,dt\\ \hphantom{dV^{i}_{t} =}{}- \sum_{ \gamma\in \mathcal {P}} (V^{i}_{t} - \overline{V}^{\alpha\gamma}) \frac{J_{t}^{i\gamma}}{N_{\gamma}} ( \sum_{j=1}^{N} {\mathbb {1}}_{\{p(j) = \gamma\}} y^{j}_{t} )\,dt + \sigma_{\alpha}\,dW^{i,V}_{t}, \\ d J^{i \gamma}_{t} = \theta_{\alpha\gamma} ( \overline{J}^{\alpha\gamma} - J^{i \gamma}_{t} )\,dt + \sigma^{J}_{\alpha\gamma}\sqrt{J^{i \gamma}_{t}}\,dB^{i\gamma}_{t} \quad \mbox{for all }\gamma\in \mathcal {P}, \\ dy^{i}_{t} = (a^{\alpha}_{r}S_{\alpha}(V^{i}_{t}) (1-y^{i}_{t}) - a^{\alpha}_{d}y^{i}_{t} )\,dt\\ \hphantom{dy^{i}_{t} =}{}+ \sqrt{ \vert a^{\alpha}_{r}S_{\alpha}(V^{i}_{t}) (1-y^{i}_{t}) + a^{\alpha}_{d}y^{i}_{t} \vert } \chi(y^{i}_{t})\,dW^{i,y}_{t}, \end{cases} $$

coupled with (2) or (3).

### The Mean-Field Limit Models

When making the $N_{\alpha}$s tend to infinity, the linear structure of the above *N*-neuron models w.r.t. the $(y^{i}_{t})$, the linear structure of the dynamics of the $(y^{i}_{t})$, and the mutual independence of the Brownian motions $(B^{i,\gamma}_{t})$, $(W^{j,y}_{t})$, lead one to mean-field dynamics. The limit processes $(V^{\alpha}_{t}, y^{\alpha}_{t}, n^{\alpha}_{t}, m^{\alpha}_{t}, h^{\alpha}_{t}, w^{\alpha}_{t}, \alpha\in \mathcal {P})$ are solutions to the SDEs (5) and (9) where $(B^{\alpha\gamma}_{t},W^{\alpha,V}_{t}, W^{\alpha,n}_{t},W^{\alpha,m}_{t},W^{\alpha,h}_{t}, \alpha\in \mathcal {P})$ denote independent Brownian motions.

#### Simple Maximum Conductance Variation

For all *α* in $\mathcal {P}$,

5 $$ \textstyle\begin{cases} dV^{\alpha}_{t} = F_{\alpha}(t, V^{\alpha}_{t} , \mathbf {q}^{\alpha}_{t})\,dt - \sum_{ \gamma\in \mathcal {P}} (V^{\alpha}_{t} - \overline{V}^{\alpha\gamma}) \overline{J}^{\alpha\gamma} \mathbb {E}[y^{\gamma}_{t}]\,dt \\ \hphantom{dV^{\alpha}_{t} =}{}- \sum_{ \gamma\in \mathcal {P}} (V^{\alpha}_{t} - \overline{V}^{\alpha\gamma}) \sigma^{J}_{\alpha\gamma} \mathbb {E}[y^{\gamma}_{t}]\,dB^{\alpha\gamma}_{t} + \sigma_{\alpha}\,dW^{\alpha,V}_{t}, \\ dy^{\alpha}_{t} = (a^{\alpha}_{r}S_{\alpha}(V^{\alpha}_{t}) (1-y^{\alpha}_{t}) - a^{\alpha}_{d}y^{\alpha}_{t} )\,dt\\ \hphantom{dy^{\alpha}_{t} =}{}+ \sqrt{ \vert a^{\alpha}_{r} S_{\alpha}(V^{\alpha}_{t}) (1-y^{\alpha}_{t}) + a^{\alpha}_{d}y^{\alpha}_{t} \vert } \chi(y^{\alpha}_{t})\,dW^{\alpha,y}_{t}, \end{cases} $$

coupled with

6 $$ dw^{\alpha}_{t} = c_{\alpha}\bigl(V^{\alpha}_{t} + a_{\alpha}- b_{\alpha}w^{\alpha}_{t}\bigr)\,dt $$

or

7 $$\begin{aligned} dx^{\alpha}_{t} ={}& \bigl( \rho_{x}\bigl(V^{\alpha}_{t}\bigr) \bigl(1-x^{\alpha}_{t}\bigr) - \zeta_{x} \bigl(V^{\alpha}_{t} \bigr)x^{\alpha}_{t} \bigr) \,dt \\ &{}+ \sqrt{ \bigl\vert \rho_{x}\bigl(V^{\alpha}_{t} \bigr) \bigl(1-x^{\alpha}_{t}\bigr) + \zeta_{x} \bigl(V^{\alpha}_{t} \bigr)x^{\alpha}_{t}\bigr\vert } \chi\bigl(x^{\alpha}_{t}\bigr)\,dW^{\alpha,x}_{t} \quad \mbox{for } x=n,m,h, \end{aligned}$$

where again $\mathbf {q}^{\alpha}_{t}$ stands for $(w^{\alpha}_{t})$ in the FitzHugh–Nagumo model and for $(n^{\alpha}_{t}, m^{\alpha}_{t},h^{\alpha}_{t})$ in the Hodgkin–Huxley model.

Notice that the diffusion coefficients of the $(y^{\alpha}_{t})$ play no role in the above mean-field dynamics since one readily sees that

8 $$\begin{aligned} \mathbb {E}\bigl[y^{\alpha}_{t}\bigr] ={}& \mathbb {E}\bigl[y_{0}^{\alpha}\bigr] \exp \biggl\{ - a^{\alpha}_{d} t - a_{r}^{\alpha}\int_{0}^{t} \mathbb {E}\bigl[S_{\alpha}\bigl(V^{\alpha}_{\theta}\bigr)\bigr]\,d \theta \biggr\} \\ & {}+ \int_{0}^{t} a_{r}^{\alpha} \mathbb {E}\bigl[S_{\alpha}\bigl(V^{\alpha}_{s}\bigr)\bigr] \\ &{}\times\exp \biggl\{ - a^{\alpha}_{d} (t-s) - a_{r}^{\alpha}\int_{s}^{t} \mathbb {E}\bigl[S_{\alpha}\bigl(V^{\alpha}_{\theta}\bigr)\bigr]\,d\theta \biggr\} \,ds\quad \mbox{for }\alpha\in \mathcal {P}. \end{aligned}$$

#### Sign-Preserving Maximum Conductance Variation

With the same notation, for all *α* in $\mathcal {P}$,

9 $$ \textstyle\begin{cases} dV^{\alpha}_{t} = F_{\alpha}(t, V^{\alpha}_{t} , \mathbf {q}^{\alpha}_{t})\,dt - \sum_{ \gamma\in \mathcal {P}} (V^{\alpha}_{t} - \overline{V}^{\alpha\gamma}) J_{t}^{\alpha\gamma} \mathbb {E}[y_{t}^{\gamma}]\,dt + \sigma_{\alpha}\,dW^{\alpha,V}_{t}, \\ d J^{\alpha\gamma}_{t} = \theta_{\alpha\gamma} ( \overline{J}^{\alpha\gamma} - J^{\alpha\gamma}_{t} )\,dt + \sigma^{J}_{\alpha\gamma} \sqrt{J^{\alpha\gamma}_{t}}\,dB^{\alpha\gamma}_{t} \quad \mbox{for all }\gamma\in \mathcal {P}, \\ dy^{\alpha}_{t} = (a^{\alpha}_{r}S_{\alpha}(V^{\alpha}_{t}) (1-y^{\alpha}_{t}) - a^{\alpha}_{d}y^{\alpha}_{t} )\,dt\\ \hphantom{dy^{\alpha}_{t} = }{}+ \sqrt{ \vert a^{\alpha}_{r} S_{\alpha}(V^{\alpha}_{t}) (1-y^{\alpha}_{t}) + a^{\alpha}_{d}y^{\alpha}_{t} \vert } \chi(y^{\alpha}_{t})\,dW^{\alpha,y}_{t}, \end{cases} $$

coupled with (6) or (7).

As in the simple maximum conductance variation case, the diffusion coefficients of the $(y^{\alpha}_{t})$ play no role in the above mean-field dynamics and $\mathbb {E}[y^{\alpha}_{t}]$ is given by (8).

### Hypotheses

Our hypotheses on the coefficients of the neuron models are the following.

#### Hypothesis 2.1

(i) *On the ion channel models*. The function *χ* is bounded Lipschitz continuous with compact support included in the interval $(0,1)$.
  The functions $\rho_{x}$, $\zeta_{x}$ are strictly positive, Lipschitz, and bounded.
(ii) *On the chemical synapse model*. The functions $S_{\alpha}$ are of the sigmoid type, that is, $S_{\alpha}(v) = C / (1 + \exp( -\lambda(v - \delta)))$ with suitable positive parameters *C*, *λ*, *δ* depending on *α*. The parameters $a^{\alpha}_{r}$, $a^{\alpha}_{d}$ are also positive.
(iii) *On the membrane model*. The drift terms $F_{\alpha}$ are continuous, one-sided Lipschitz w.r.t. *v* and Lipschitz w.r.t. **q**. More precisely, there exist a positive real number *L* and a positive map $M(v,v')$ such that for all $t\in[0,T]$, for all **q**, $\mathbf {q}'$ in $\mathbb {R}^{3}$ or $\mathbb {R}$, *v*, $v'$ in $\mathbb {R}$,
  10 $$ \begin{aligned} \bigl(F_{\alpha}(t,v,\mathbf {q}) - F_{\alpha}\bigl(t,v',\mathbf {q}\bigr)\bigr) \bigl(v-v' \bigr) &\leq L\bigl(v - v'\bigr)^{2} - M \bigl(v,v'\bigr) \bigl(v - v'\bigr)^{2}, \\ \bigl\vert F_{\alpha}(t,v,\mathbf {q}) - F_{\alpha}\bigl(t,v, \mathbf {q}'\bigr)\bigr\vert &\leq L\bigl\Vert \mathbf {q}-\mathbf {q}' \bigr\Vert . \end{aligned} $$
(iv) *The initial conditions*. $V^{i}_{0}$, $J^{i\gamma}_{0}$, $y^{i}_{0}$, $w^{i}_{0}$, $n^{i}_{0}$, $m^{i}_{0}$, $h^{i}_{0}$ are i.i.d. random variables with the same law as $V^{\alpha}_{0}$, $J^{\alpha\gamma}_{0}$, $y^{\alpha }_{0}$, $w^{\alpha}_{0}$, $n^{\alpha}_{0}$, $m^{\alpha}_{0}$, $h^{\alpha}_{0}$, when $p(i)=\alpha$. We also assume that $V^{\alpha}_{0}$ and $J^{\alpha\gamma}_{0}$ admit moments of any order.
  Moreover, the support of the law of $y^{\alpha}_{0}$ belongs to $[0,1]$, as well as the support of the laws of $n^{\alpha}_{0}$, $m^{\alpha}_{0}$, $h^{\alpha}_{0}$, for all *α* in $\mathcal {P}$. The support of the law of $J^{\alpha\gamma}_{0}$ belongs to $(0,+\infty)$.

#### Remark 2.2

For each neuron population *α*, the function $S_{\alpha}$ represents the concentrations of the transmitter released into the synaptic cleft by a presynaptic spike.

Our hypothesis on the support of the function *χ* is essential to force the proportion processes $(y^{i}_{t})$, $(n^{i}_{t})$, $(m^{i}_{t})$, $(h^{i}_{t})$ to live in the interval $(0,1)$.

In the case of the FitzHugh–Nagumo model, for all *α* we have $F_{\alpha}(v,\mathbf {q}) = - \frac{1}{3} v^{3} + v -w$, so that we may choose $L=1$ and $M(v,v') = \frac{1}{3} (\vert v\vert - \vert v'\vert )^{2}$.

Finally, the i.i.d. hypothesis in part (iv) is only used in Sect. 4 where it allows simplifications, but it can be relaxed to initial chaos by classical arguments in the propagation of chaos literature.

#### Remark 2.3

We notice that a one-sided Lipschitz condition also appears in the work by Luçon and Stannat [7] for stochastic particle systems in random environments in which they model one-population networks of FitzHugh–Nagumo neurons. However, their model does not include synaptic interactions as ours does. This has led us in particular to consider square root diffusion coefficients in the dynamics of the synaptic variables which, as shown below, requires specific arguments to prove that the particle systems are well posed and propagate chaos.

#### Remark 2.4

The boundedness of the functions $\rho_{x}$ and $\zeta_{x}$ is a technical hypothesis which simplifies the analysis but can be relaxed, provided that the above models have solutions which do not explode in finite time. However, this comfortable hypothesis is quite reasonable for neuron models since the membrane potentials take values between −100 mV and 100 mV. It is therefore implicitly understood that Lipschitz functions which reasonably fit data within the interval $[-100,100]$ are extended to bounded Lipschitz functions on the entire real line.

## SDEs in Rectangular Cylinders

In the above *N*-neuron and limit models one requires that, for all *i*, *α* and $x=n,m,h$, the concentration processes $(x^{i}_{t})$, $(x^{\alpha}_{t})$, $(y^{i}_{t})$ and $(y^{\alpha}_{t})$ are well defined and take values in the interval $[0,1]$. Each one of these processes is one-dimensional but not Markov since the coefficients of their dynamics depend on $(V_{t})$ and thus on all the components of the systems (4) and (1). In this context, classical arguments for one-dimensional Markov diffusion processes such as Feller’s tests or comparison theorems cannot be used to show that the processes do not exit from $[0,1]$. We thus need to develop an ad hoc technique. Instead of focusing on the above neuron models, we rather introduce a more general setting.

Consider the stochastic differential equation

11 $$ \textstyle\begin{cases} dX^{(1)}_{t} = b_{1}(X_{t},U_{t})\,dt+A_{1}(X_{t},U_{t})\,dW^{(1)}_{t}, \\ \ldots \\ dX^{(k)}_{t} = b_{k}(X_{t},U_{t})\,dt+A_{k}(X_{t},U_{t})\,dW^{(k)}_{t}, \\ dU_{t} = \beta(X_{t},U_{t})\,dt+\varGamma(X_{t},U_{t})\,dW^{\ast}_{t}, \end{cases} $$

where $(X_{t}) := (X^{(1)}_{t},\ldots,X^{(k)}_{t})$ is $\mathbb {R}^{k}$-valued, $(U_{t})$ is $\mathbb {R}^{d}$-valued, $(W_{t})= (W^{(1)}_{t},\ldots, W^{(k)}_{t})$ is a $\mathbb {R}^{k}$-valued $(\mathcal{F}_{t})$-Brownian motion, and $(W^{\ast}_{t})$ is a $\mathbb {R}^{r}$-valued Brownian motion.

We aim to exhibit conditions on the coefficients of (11) which imply that the process $(X_{t},U_{t})$ takes values in the infinite rectangular cylinder $[0,1]^{k}\times \mathbb {R}^{d}$.

### Remark 3.1

Many stochastic models of the type (11) which arise in physics need to satisfy the constraint that the process $(X_{t})$ is valued in the hypercube, say, $[0,1]^{k}$. The algebraic expressions of the coefficients derived from physical laws may be ‘naturally’ defined only for *x* in $[0,1]^{k}$. However, one typically can construct continuous extensions of these coefficients on the whole Euclidean space. These extensions may be arbitrarily chosen, provided that they satisfy Hypothesis 3.2 and that Eq. (11) has a weak solution which does not explode in finite time. In Sect. 4 we develop this argument to show that all systems in Sect. 2 are well posed and that the concentration processes are all valued in $[0,1]$.

Assume the following.

### Hypothesis 3.2

The locally Lipschitz continuous functions $b_{i}$, $A_{i}$, *β* and *Γ* are such that, on some filtered probability space $(\varOmega,\mathcal{F},\mathbb{P},(\mathcal{F}_{t},t\geq0))$, there exists a weak solution to (11) which does not explode in finite time. In addition:

(i) The functions $A_{i}$, $i=1,\ldots,k$, satisfy, for all *u* in $\mathbb {R}^{d}$,
  $$\forall x_{i}\in \mathbb {R}\setminus(0,1),\quad A_{i}(x_{1}, \ldots,x_{i-1},x_{i},x_{i+1},\ldots ,x_{k},u)=0. $$
(ii) The functions $b_{i}$, $i=1,\ldots,k$, satisfy, for all *u* in $\mathbb {R}^{d}$ and $x_{1},\ldots,x_{i-1},x_{i+1},\ldots, x_{k}$ in $\mathbb {R}^{k-1}$,
  $$ \textstyle\begin{cases} b_{i}(x ,u)\geq0 &\mbox{on }\{x_{i}\leq0\}, \\ b_{i}(x,u)\leq0 &\mbox{on }\{x_{i} \geq1\}. \end{cases} $$

The following argument implies that we may limit ourselves to the case $k=1$. Set $U^{\sharp}:=(X^{(2)},\ldots,X^{(k)},U)$. Then, for obviously defined new coefficients $\beta_{1}^{\sharp}$, $A^{\sharp}$, etc., and a $\mathbb {R}^{r+k-1}$-valued Brownian motion $W^{\ast\sharp}$ one has

$$ \textstyle\begin{cases} dX^{(1)}_{t} = b^{\sharp}_{1}(X^{(1)}_{t},U^{\sharp}_{t})\,dt +A^{\sharp}_{1}(X^{(1)}_{t},U^{\sharp}_{t})\,dW^{(1)}_{t}, \\ dU^{\sharp}_{t} = \beta^{\sharp}_{1}(X^{(1)}_{t},U^{\sharp}_{t})\,dt +\varGamma^{\sharp}_{1}(X^{(1)}_{t},U^{\sharp}_{t})\,dW^{\ast\sharp}_{t}. \end{cases} $$

If we can prove that $X^{(1)}$ takes values in $[0,1]$, then the same arguments would show that all the other components enjoy the same property. We therefore consider the system (11) with $k=1$.

### Proposition 3.3

*Suppose that*$0\leq X^{(1)}_{0} \leq1$*a*.*s*. *Under Hypothesis*3.2

$$\mathbb{P} \bigl( \forall t \geq0, 0 \leq X_{t}^{(1)} \leq1 \bigr) = 1. $$

### Proof

We limit ourselves to the proof that $0\leq X_{t}^{(1)}$ for all $t\geq 0$ a.s. We can use very similar arguments to get the other inequality.

Let $\varPsi_{\epsilon}$ be a positive decreasing function of class $\mathcal{C}^{2}(\mathbb {R})$ with $\varPsi_{\epsilon}(x)=1$ on $(-\infty,-\epsilon]$ and $\varPsi _{\epsilon}(x)=0$ on $[0,+\infty)$. Let $\tau_{n}$ be the first time the process $(X^{(1)}_{t})$ exits from the interval $(-n,n)$. As $\varPsi_{\epsilon}(x)=0$ on $\mathbb {R}_{+}$, $\varPsi_{\epsilon}(X^{(1)}_{0})=0$ and Itô’s formula leads to

$$\begin{aligned} \varPsi_{\epsilon}\bigl(X^{(1)}_{t\wedge\tau_{n}}\bigr) =& \int _{0}^{t\wedge\tau_{n}} {\mathbb {1}}_{\{X^{(1)}_{s} \leq0\}} b^{\sharp}_{1}\bigl(X^{(1)}_{s},U^{\sharp}_{s} \bigr) \varPsi_{\epsilon}'\bigl(X^{(1)}_{s} \bigr)\,ds \\ &{} +\frac{1}{2} \int_{0}^{t\wedge\tau_{n}} {\mathbb {1}}_{ \{X^{(1)}_{s} \leq0\}} \bigl(A^{\sharp}_{1} \bigl(X^{(1)}_{s},U^{\sharp}_{t}\bigr) \bigr)^{2} \varPsi_{\epsilon}'' \bigl(X^{(1)}_{s}\bigr)\,ds \\ &{} + \int_{0}^{t\wedge\tau_{n}} {\mathbb {1}}_{ \{X^{(1)}_{s}\leq0\}} A^{\sharp}_{1} \bigl(X^{(1)}_{s},U^{\sharp}_{s}\bigr) \varPsi_{\epsilon}'\bigl(X^{(1)}_{s}\bigr) \,dW^{\ast\sharp}_{s} \\ =:& I_{1} + I_{2} + I_{3}. \end{aligned}$$

As $\varPsi_{\epsilon}$ is decreasing, Hypothesis 3.2(ii) and the definition of $b^{\sharp}_{1}$ imply that $I_{1}\leq0$. As ${\mathbb {1}}_{\{ x\leq0\}}A^{\sharp}_{1}(x,u)=0$ for all $(x,u)$, one has $I_{2}=0$. Finally, $I_{3}$ is a martingale. Therefore

$$\forall t>0,\quad \mathbb {E}\varPsi_{\epsilon}\bigl(X^{(1)}_{t\wedge\tau_{n}} \bigr)=0. $$

Fatou’s lemma implies

$$\forall t>0,\quad \mathbb {E}\varPsi_{\epsilon}\bigl(X^{(1)}_{t} \bigr)=0. $$

Now consider a family of functions $\varPsi_{\epsilon}$ which pointwise converge to ${\mathbb {1}}_{(-\infty,0)}$ and satisfy $\sup_{\epsilon} \vert \varPsi_{\epsilon} \vert _{\infty}=1$. Lebesgue’s dominated convergence theorem implies

$$\forall t>0, \quad \mathbb {E}\bigl[{\mathbb {1}}_{(-\infty,0)}\bigl(X^{(1)}_{t} \bigr)\bigr]=0. $$

In other words, the process $((X_{t}^{(1)})^{-},t\geq0)$ is a modification of the null process. As they both are continuous processes, they are indistinguishable (see, e.g., Karatzas and Shreve [8], Chap. 1, Pb. 1.5). □

## The Models Are Well-Posed Diffusions in Rectangular Cylinders and Propagate Chaos

In this section, we check that the particle systems and the mean-field limit systems are well posed, and that the components of the processes $(y^{i}_{t})$, $(x^{i}_{t})$, $(y^{\alpha}_{t})$, $(x^{\alpha}_{t})$ take values in $[0,1]$. Then we prove that the particle systems propagate chaos toward the law of the limit systems (5) and (9).

Our situation differs from the above mentioned Scheutzow’s counterexamples [4] in the fact that the interaction kernel is globally Lipschitz and the functions $F_{\alpha}$ are one-sided Lipschitz (they are not only locally Lipschitz). These features of the neuronal models under consideration protect one from non-uniqueness of the solutions.

### Well-Posedness of the *N*-Neuron Models

#### Proposition 4.1

*Under Hypothesis*2.1*the systems* (1) *and* (4) *have unique pathwise solutions on all time interval*$0\leq t\leq T$. *In addition*, *the components of the processes*$(y^{i}_{t})$, $(n^{i}_{t})$, $(m^{i}_{t})$, $(h^{i}_{t})$*take values in*$[0,1]$.

#### Proof

Observe that the coefficients of (1) and (4) are locally Lipschitz. This is obvious for the drift coefficients. In view of the assumption on the function *χ*, the diffusion coefficients obviously are locally Lipschitz at all point $(v,x)$ (respectively, $(v,y)$) with *x* or *y* in $\mathbb {R}\setminus[0,1]$; this also holds true at all the other points since, for all $\lambda_{1}>0$ and $\lambda_{2}>0$, the value of *z* such that $\lambda_{1}(1-z)+\lambda_{2} z=0$ never belongs to $[0,1]$, so that the arguments of the square roots in the diffusion coefficients are strictly positive when *x* (respectively, *y*) belongs to $[0,1]$.

It results from the preceding observation that solutions to (1) and (4) exist and are pathwise unique up to their respective explosion times: see, e.g., Protter [9], Chap. V, Sect. 7, Theorem 38. Set

$$\xi^{i}_{t} := \bigl(V^{i}_{t},y^{i}_{t}, \mathbf {q}^{i}_{t}\bigr) \quad \text{or}\quad \bigl(V^{i}_{t},J^{i\gamma }_{t},y^{i}_{t}, \mathbf {q}^{i}_{t}\bigr), \quad \gamma\in \mathcal {P}. $$

Using the one-sided Lipschitz condition (10) and Itô’s formula, it is an easy exercise to see that $\mathbb {E}\vert \xi^{i}_{T}\vert ^{2}$ is finite for all $T>0$, from which it readily follows that $\mathbb {E}\sup_{0\leq t\leq T}\vert \xi^{i}_{t}\vert ^{2}$ is finite for all $T>0$. Therefore the explosion times of (1) and (4) are infinite a.s.

Let us now check that the coordinates of $(y^{i}_{t},n^{i}_{t},m^{i}_{t}, h^{i}_{t})$ take values in $[0,1]$. Their dynamics is of the type

12 $$ \begin{aligned}[b] dx_{t} = {}& \bigl( \rho_{x}(V_{t}) (1-x_{t}) - \zeta_{x}(V_{t} )x_{t} \bigr)\,dt \\ &{}+ \sqrt{\bigl\vert \rho_{x}(V_{t}) (1-x_{t}) + \zeta_{x}(V_{t} )x_{t}\bigr\vert } \chi(x_{t})\,dW^{x}_{t} \end{aligned} $$

for $x= n, m, h$, and

13 $$ \begin{aligned}[b] dy^{\alpha}_{t} ={}& \bigl(a^{\alpha}_{r}S_{\alpha}(V_{t}) \bigl(1-y^{\alpha}_{t}\bigr) - a^{\alpha}_{d}y^{\alpha}_{t} \bigr)\,dt \\ &{}+ \sqrt{ \bigl\vert a^{\alpha}_{r} S_{\alpha}(V_{t}) \bigl(1-y^{\alpha}_{t}\bigr) + a^{\alpha}_{d}y^{\alpha}_{t} \bigr\vert } \chi \bigl(y^{\alpha}_{t}\bigr)\,dW^{\alpha,y}_{t}, \end{aligned} $$

where $V_{t}$ is some real-valued continuous process. Hypothesis 3.2(ii) is satisfied by the drift coefficients of (12) and (13):

$$\begin{aligned} (x,v) &\mapsto b_{x}(x,v) := \rho_{x}(v) (1-x) - \zeta_{x}(v)x\quad \mbox{and} \quad \\ (y,v) &\mapsto b_{y}(y,v) := a_{r} ^{\alpha}S_{\alpha}(v) (1-y) - a_{d}^{\alpha} y. \end{aligned}$$

The desired result follows, by applying Proposition 3.3. □

#### Remark 4.2

The preceding discussion shows that one can get rid of the absolute values in the diffusion coefficients of all the models in Sect. 2.

### Well-Posedness of the Mean-Field Limit Models

For the next proposition we slightly reinforce the hypotheses on the functions $\rho_{x}$ and $\zeta_{x}$. The boundedness from below by a strictly positive constant is justified from a biological point of view (see the discussion in [1], Sect. 2.1, p. 5).

#### Proposition 4.3

*Under Hypothesis*2.1*and the coercivity condition*

14 $$\begin{aligned} \exists\nu>0, \forall v\in \mathbb {R}, \quad \rho_{x}(v)\wedge \zeta_{x}(v)\geq\nu>0, \end{aligned}$$

*the systems* (5) *and* (9), *complemented with* (6) *or* (7), *have unique pathwise solutions on all time interval*$[0,T]$. *In addition*, *all the components of the process*$(y^{\alpha}_{t}, n^{\alpha}_{t},m^{\alpha}_{t},h^{\alpha}_{t})$*take values in*$[0,1]$.

#### Proof

Existence and pathwise uniqueness are obtained by slightly extending the fixed point method developed by Sznitman [2] for particle systems with bounded Lipschitz coefficients. We essentially combine arguments already available in the literature (e.g. see [7] and references therein) and therefore only emphasize the additional minor arguments required by the above neuron models. As exactly the same arguments can be used to treat Eqs. (5) and (9), we limit ourselves to consider the second one.

We start with the following observation. Given the Brownian motions $(B^{\alpha\gamma}, \alpha, \gamma\in \mathcal {P})$ and the constant $\overline{J}^{\alpha\gamma}$, the processes $(J^{\alpha \gamma}_{t},\alpha,\gamma\in \mathcal {P})$ are unique pathwise solutions according to the Yamada and Watanabe theorem (see, e.g., Karatzas and Shreve [8], Chap. 5, Theorem 2.13). Let $\varphi(t)$ be an arbitrary continuous function. Consider the system obtained by substituting $\varphi(t)$ for $\mathbb {E}[y_{t}^{\gamma}]$ into (9). Similar arguments as in the proof of Proposition 4.1 show that this new system has a unique pathwise solution.

Now, we denote by $\ell=\overline{p}^{2}+6\overline{p}$ the dimension of the state space of the process

$$\bigl(V^{\alpha}_{t},J^{\alpha\gamma}_{t},y^{\alpha}_{t}, w^{\alpha}_{t},\bigl(n^{\alpha}_{t},m^{\alpha}_{t},h^{\alpha}_{t} \bigr); \alpha,\gamma\in \mathcal {P}\bigr). $$

#### Remark 4.4

Notice that we have lumped together the two models we are focusing on, i.e. FitzHugh–Nagumo and Hodgkin–Huxley, since the mathematical tools for handling them are similar: Hypothesis 2.1(iii) does not hold for the classic Hodgkin–Huxley model. However, well-posedness and propagation of chaos can be obtained in this case by adding standard localisation arguments to our proofs.

Let

$$(r_{t}) :=\bigl(v^{\alpha}_{t},j^{\alpha\gamma}_{t}, \psi^{\alpha}_{t},w^{\alpha}_{t}, z^{\alpha}_{t}; \alpha,\gamma\in \mathcal {P}\bigr) $$

be the canonical process of $\mathcal{C}(0,T;\mathbb {R}^{\ell})$. Let $\mathcal{C}_{T} $ be the subspace of $\mathcal{C}(0,T;\mathbb {R}^{\ell})$ consisting of the paths of the canonical process such that $(\psi^{\alpha}_{t},z^{\alpha}_{t})$ takes values in $[0,1]^{4}$ for all *t*, $\alpha\in \mathcal {P}$.

Equip the space $\mathcal{M}_{T}$ of probability measures on $\mathcal{C}_{T}$ with the standard Wasserstein (2) metric:

$$\mathbb {W}_{T}(\pi_{1},\pi_{2}) := \biggl\{ \min _{\mu\in\varLambda(\pi_{1},\pi_{2})} \int_{C_{T} } \sup_{0\leq s\leq T} \bigl( \bigl\vert r^{1}_{s}-r^{2}_{s} \bigr\vert ^{2} \bigr) \mu\bigl(dr^{1},dr^{2} \bigr) \biggr\} ^{1/2}, $$

where $\varLambda(\pi_{1},\pi_{2})$ denotes the set of all coupling measures of $\pi_{1}$ and $\pi_{2}$.

Given *π* in $\mathcal{M}_{T}$, set

$$\begin{aligned} \overline{y}^{(\pi ),\gamma}_{t} := {}&\mathbb {E}_{\pi}\bigl[y_{0}^{\gamma}\bigr] \exp \biggl\{ - a^{\gamma}_{d} t - a_{r}^{\gamma}\int_{0}^{t} \mathbb {E}_{\pi}\bigl[S_{\gamma}\bigl(v^{\gamma}_{\theta}\bigr)\bigr]\,d\theta \biggr\} \\ &{} + \int_{0}^{t} a_{r}^{\gamma} \mathbb {E}_{\pi}\bigl[S_{\gamma}\bigl(v^{\gamma}_{s} \bigr)\bigr] \\ &{}\times\exp \biggl\{ - a^{\gamma}_{d} (t-s) - a_{r}^{\gamma}\int_{s}^{t} \mathbb {E}_{\pi}\bigl[S_{\gamma}\bigl(v^{\gamma}_{\theta}\bigr)\bigr]\,d\theta \biggr\} \,ds\quad \mbox{for all }\gamma\in \mathcal {P}. \end{aligned}$$

Let us construct a contraction map *Φ* on $\mathcal{M}_{T}$ as follows. For all *π* in $\mathcal{M}_{T}$, $\varPhi(\pi)$ is the probability law of the process

$$\bigl(R^{(\pi )}_{t}\bigr) :=\bigl(V^{(\pi ),\alpha}_{t},J^{\alpha\gamma}_{t},y^{(\pi ),\alpha }_{t},w^{(\pi ),\alpha}_{t}, x^{(\pi ),\alpha}_{t}; \alpha,\gamma\in \mathcal {P}; x=n,m,h\bigr) $$

solution to

$$\begin{aligned} dV^{(\pi ),\alpha}_{t} ={}& F_{\alpha}\bigl(t, V^{(\pi ),\alpha}_{t} , \mathbf {q}^{(\pi ),\alpha }_{t}\bigr)\,dt \\ &{}- \sum_{ \gamma\in \mathcal {P}} \bigl(V^{(\pi ),\alpha}_{t} - \overline{V}^{\alpha\gamma}\bigr) J_{t}^{\alpha\gamma} \overline{y}^{(\pi ),\gamma}_{t} \,dt + \sigma_{\alpha}\,dW^{\alpha_{t},V}, \\ d J^{\alpha\gamma}_{t} ={}& \theta_{\alpha\gamma} \bigl( \overline{J}^{\alpha\gamma} - J^{\alpha\gamma}_{t} \bigr)\,dt + \sigma^{J}_{\alpha\gamma} \sqrt{J^{\alpha\gamma}_{t}} \,dB^{\alpha\gamma}_{t}, \\ dy^{(\pi ),\alpha}_{t} ={}& \bigl(a^{\alpha}_{r} S_{\alpha}\bigl(V^{(\pi ),\alpha }_{t}\bigr) \bigl(1-y^{(\pi ),\alpha}_{t} \bigr) - a^{\alpha}_{d} y^{(\pi ),\alpha}_{t} \bigr)\,dt \\ &{}+ \sqrt{ \bigl\vert a^{\alpha}_{r} S_{\alpha}\bigl(V^{(\pi ),\alpha}_{t}\bigr) \bigl(1-y^{(\pi ),\alpha}_{t} \bigr) + a^{\alpha}_{d} y^{(\pi ),\alpha}_{t} \bigr\vert } \chi\bigl(y^{(\pi ),\alpha}_{t}\bigr)\,dW^{\alpha,y}_{t}, \\ dw^{(\pi ),\alpha}_{t} ={}& c_{\alpha}\bigl(V^{(\pi ),\alpha}_{t} + a_{\alpha}- b_{\alpha}w^{(\pi ),\alpha}_{t}\bigr)\,dt, \\ dx^{(\pi ),\alpha}_{t} ={}& \bigl( \rho_{x} \bigl(V^{(\pi ),\alpha}_{t}\bigr) \bigl(1-x^{(\pi ),\alpha}_{t} \bigr) - \zeta_{x}\bigl(V^{(\pi ),\alpha}_{t} \bigr)x^{(\pi ),\alpha}_{t} \bigr)\,dt \\ & {}+ \sqrt{\bigl\vert \rho_{x}\bigl(V^{(\pi ),\alpha}_{t} \bigr) \bigl(1-x^{(\pi ),\alpha}_{t}\bigr) + \zeta_{x} \bigl(V^{(\pi ),\alpha}_{t} \bigr)x^{(\pi ),\alpha}_{t}\bigr\vert } \chi\bigl(x^{(\pi ),\alpha}_{t}\bigr)\,dW^{\alpha,x}_{t} \end{aligned}$$

with $x=n,m,h$, $\mathbf {q}=(w)$ or $\mathbf {q}=(x)$. Notice that the probability law of the process $(R^{(\pi )}_{t})$ is supported in $\mathcal{C}_{T}$.

Set $\Delta R_{t} := R^{(\pi_{1})}_{t} - R^{(\pi_{2})}_{t}$, and apply Itô’s formula to $(\Delta R_{t})^{2}$. In order to deduce that there exists a positive constant $K_{T}$ uniform w.r.t. $\pi_{1}$ and $\pi_{2}$ such that

15 $$ \forall0\leq t\leq T,\quad \mathbb {E}\vert \Delta R_{t} \vert ^{2} \leq K_{T} \biggl( \int_{0}^{t} \mathbb {E}\vert \Delta R_{s}\vert ^{2}\,ds + \int _{0}^{t} \mathbb {W}_{s}(\pi_{1}, \pi_{2})\,ds \biggr), $$

it suffices to use classical arguments, plus the following ingredients:

- The one-sided Lipschitz condition (10);
- the fact that $\overline{y}^{(\pi ),\alpha}_{t}$ is uniformly bounded w.r.t. *π* in $\mathcal{M}_{T}$ and *t* in $[0,T]$;
- the additional coercivity condition (14) implies
  $$\forall x\in[0,1], \forall v\in \mathbb {R},\quad \rho_{x}(v) (1-x) + \zeta_{x}(v) x \geq\nu>0. $$
  As $(x^{(\pi_{1}),\alpha}_{t})$ and $(x^{(\pi_{2}),\alpha}_{t})$ take values in $[0,1]$, all the terms of the type
  16 $$\begin{aligned} &\int_{0}^{t} \Bigl( \sqrt {\bigl\vert \rho_{x}\bigl(V^{(\pi_{1}),\alpha}_{s} \bigr) \bigl(1-x^{(\pi_{1}),\alpha}_{s}\bigr) + \zeta_{x} \bigl(V^{(\pi_{1}),\alpha}_{s}\bigr)x^{(\pi_{1}),\alpha}_{s}\bigr\vert } \chi \bigl(x^{(\pi _{1}),\alpha}_{s}\bigr) \\ &\quad {}- \sqrt{\bigl\vert \rho_{x}\bigl(V^{(\pi_{2}),\alpha}_{s} \bigr) \bigl(1-x^{(\pi_{2}),\alpha}_{s}\bigr) + \zeta_{x} \bigl(V^{(\pi_{2}),\alpha}_{s}\bigr)x^{(\pi_{2}),\alpha}_{s}\bigr\vert }\chi \bigl(x^{(\pi _{2}),\alpha}_{s}\bigr) \Bigr)^{2}\,ds \end{aligned}$$
  are bounded from above by
  $$K_{T}\int_{0}^{t} \bigl\vert x^{(\pi_{1}),\alpha}_{s}-x^{(\pi_{2}),\alpha}_{s} \bigr\vert ^{2}\,ds + K_{T}\int_{0}^{t} \bigl\vert V^{(\pi_{1}),\alpha}_{s}-V^{(\pi_{2}),\alpha}_{s} \bigr\vert ^{2}\,ds $$
  (the same remarks apply to the diffusion coefficients of $(y^{(\pi _{1}),\alpha}_{t})$ and $(y^{(\pi_{2}),\alpha}_{t})$);
- the existence of a positive $K_{T}$ uniform w.r.t. $\pi_{1}$ and $\pi _{2}$ such that, for all $\alpha\in \mathcal {P}$,
  $$\sup_{0\leq t\leq T}\bigl\vert \overline{y}^{(\pi_{1}),\alpha}_{t} - \overline{y}^{(\pi_{2}),\alpha}_{t} \bigr\vert ^{2} \leq K_{T} \int_{0}^{T} \mathbb {W}^{2}_{s}(\pi_{1},\pi_{2})\,ds. $$

Classical arguments allow one to deduce from (15) that, for some possibly new positive constant $K_{T}$,

$$\mathbb {W}^{2}_{T}\bigl(\varPhi(\pi_{1}),\varPhi( \pi_{2})\bigr) \leq K_{T} \int_{0}^{T} \mathbb {W}^{2}_{s}(\pi_{1},\pi_{2})\,ds. $$

One then finds that *Φ* is a contraction map (see Sznitman [2]), from which the desired existence and pathwise uniqueness result follows for (9).

It remains to again use Proposition 3.3 to get the last part in the statement.  □

### Convergence

In this part, we analyze the convergence of the *N*-neurons system given in (4) to the mean-field limit described in (9). The convergence of the model (1) to the solution of (5) results from a straightforward adaptation of the following proposition and of its proof. Moreover, as in the proof of Proposition 4.3 we use again Remark 4.4 to shorten the presentation.

Let $(R_{t}) = (R^{\alpha}_{t},\alpha\in \mathcal {P}) = (V^{\alpha}_{t},(J^{\alpha \gamma }_{t},\gamma\in \mathcal {P}), y^{\alpha}_{t}, w^{\alpha}_{t},n^{\alpha}_{t}, m^{\alpha}_{t}, h^{\alpha}_{t};\alpha\in \mathcal {P})$ denote the solution of (9), with law $\mathbb{P}$ on $C_{T}$. Let $(R^{i,N}_{t},i=1,\ldots,N)= (V^{i}_{t},(J^{i\gamma}_{t}, \gamma\in \mathcal {P}), y^{i}_{t}, w^{i}_{t},n^{i}_{t}, m^{i}_{t},h^{i}_{t};i=1,\ldots,N)$, the solution of the *N*-neuron system (4). Considering the family of Brownian motions in (4), and the set of i.i.d. initial random variables $(V^{i}_{0},(J^{i\gamma}_{0}, \gamma\in \mathcal {P}), y^{i}_{0}, w^{i}_{0},n^{i}_{0},m^{i}_{0},h^{i}_{0};i=1,\ldots,N)$, we introduce a coupling between the $(R^{i,N}_{t},i=1,\ldots,N)$ and a set of *N* processes $({{\widetilde {R}}}^{i}_{t},i=1,\ldots,N)$ such that for all $\alpha\in \mathcal {P}$, all $N_{\alpha}$ indices *i* such that $p(i)=\alpha$ are such that $({{\widetilde {R}}}^{i}_{t})$ are independent copies of $(R^{\alpha}_{t})$. More precisely, for each $i=1,\ldots,N$, such that $p(i)=\alpha$, $({{\widetilde {R}}}^{i}_{t})= (\widetilde{V}^{i}_{t},(\widetilde{J}^{i\gamma}_{t}, \gamma\in \mathcal {P}),\widetilde{y}^{i}_{t}, \widetilde{w}^{i}_{t},\widetilde{n}^{i}_{t},\widetilde {m}^{i}_{t},\widetilde{h}^{i}_{t})$ is the solution of

$$\begin{aligned} \textstyle\begin{cases} \mbox{for }\widetilde {\mathbf {q}}= (\widetilde{w}) \mbox{ or } (\widetilde {n},\widetilde{m},\widetilde{h}), \\ d\widetilde{V}^{i}_{t} = F_{\alpha}(t, \widetilde{V}^{i}_{t} , \widetilde {\mathbf {q}}^{i}_{t})\,dt - \sum_{ \gamma\in \mathcal {P}} (\widetilde{V}^{i}_{t} - \overline {V}^{\alpha \gamma}) \widetilde{J}_{t}^{i\gamma} \mathbb {E}[y_{t}^{\gamma}]\,dt + \sigma_{\alpha}\,dW^{i,V}_{t}, \\ (\widetilde{J}^{i \gamma}_{t}, t\geq0) = (J^{i \gamma}_{t}, t\geq 0)\quad \text{for all }\gamma\in \mathcal {P}, \\ d\widetilde{y}^{i}_{t} = (a^{\alpha}_{r}S_{\alpha}(\widetilde{V}^{i}_{t}) (1-\widetilde{y}^{i}_{t}) - a^{\alpha}_{d}\widetilde{y}^{i}_{t} )\,dt\\ \hphantom{d\widetilde{y}^{i}_{t} =}{}+ \sqrt{ \vert a^{\alpha}_{r}S_{\alpha}(\widetilde{V}^{i}_{t}) (1-\widetilde{y}^{i}_{t}) + a^{\alpha}_{d}\widetilde{y}^{i}_{t} \vert } \chi(\widetilde{y}^{i}_{t})\,dW^{i,y}_{t}, \end{cases}\displaystyle \end{aligned}$$

coupled with

$$\begin{aligned} d\widetilde{w}^{i}_{t} = c_{\alpha}\bigl( \widetilde{V}^{i}_{t} + a_{\alpha}- b_{\alpha}\widetilde{w}^{i}_{t}\bigr)\,dt \end{aligned}$$

or

$$ \begin{aligned} d\widetilde{x}^{i}_{t} ={}& \bigl( \rho_{x} \bigl(\widetilde{V}^{i}_{t}\bigr) \bigl(1- \widetilde{x}^{i}_{t}\bigr) - \zeta_{x}\bigl( \widetilde{V}^{i}_{t}\bigr)\widetilde{x}^{i}_{t} \bigr)\,dt \\ &{} + \sqrt{\bigl\vert \rho_{x}\bigl( \widetilde{V}^{i}_{t}\bigr) \bigl(1-\widetilde{x}^{i}_{t} \bigr) + \zeta_{x}\bigl(\widetilde{V}^{i}_{t} \bigr) \widetilde{x}^{i}_{t}\bigr\vert } \chi\bigl( \widetilde{x}^{i}_{t}\bigr)\,dW^{i,x}_{t} \end{aligned} $$

for $\widetilde{x}=\widetilde{n},\widetilde{m},\widetilde{h}$, and starting at $(V^{i}_{0},(J^{i\gamma}_{0}, \gamma\in \mathcal {P}), y^{i}_{0}, w^{i}_{0},n^{i}_{0},m^{i}_{0},h^{i}_{0})$.

Under the hypotheses of Proposition 4.3 we have the following result as regards the propagation of chaos.

#### Proposition 4.5

*Assume that for all*$\gamma\in \mathcal {P}$, *the proportion*$N_{\gamma}/N$*of neurons in populationγis a nonzero constant independent ofNand denoted*:

17 $$\begin{aligned} c_{\gamma}= \frac{N_{\gamma}}{N}. \end{aligned}$$

*Then there exists a constant*$C>0$*such that*, *for all*$N = \sum_{\gamma \in \mathcal {P}} N_{\gamma}$*satisfying the assumption* (17), *for all set ofp̅indices*$(i_{\alpha}, \alpha\in \mathcal {P})$*among*$\{ 1,\ldots,N\}$*with*$p(i_{\alpha}) = \alpha$, *the vector process*$(R^{i_{\alpha},N} - {{\widetilde {R}}}^{i_{\alpha}})$, *with one component in each population*, *satisfies*

18 $$ \sqrt{N} \mathbb {E}\biggl[ \sup_{0\leq t\leq T} \sum _{\alpha\in \mathcal {P}} \bigl\vert R^{i_{\alpha},N}_{t}-{ {\widetilde {R}}}^{i_{\alpha}}_{t}\bigr\vert ^{2} \biggr] \leq C. $$

The above $L^{2}(\varOmega)$-estimate obviously implies that the law of any subsystem of size *k*

$$\bigl(\bigl(R^{1_{\alpha},N}_{t},\alpha\in \mathcal {P}\bigr),\ldots, \bigl(R^{k_{\alpha},N}_{t},\alpha \in \mathcal {P}\bigr) \bigr), $$

with $p(i_{\alpha}) = \alpha$, converges to the law $\mathbb {P}^{\otimes k}$ when the $N_{\alpha}$ tend to ∞. In other words, the reordered system

$$\bigl(\bigl(R^{i_{\alpha},N}_{t},\alpha\in \mathcal {P}\bigr), p(i_{\alpha}) = \alpha, i_{\alpha}\in\{1,\ldots,N\} \bigr) $$

is $\mathbb{P}$-chaotic.

#### Proof of Proposition 4.5

*A short discussion of our methodology*. We only present the main ideas of the proof which follows and adapts Sznitman [2] or Méléard [10]. To help the reader follow the lengthy calculations, we start with an explanation of the main differences between our problem where some of the coefficients of our stochastic differential equations are not globally Lipschitz continuous and the classical Lipschitz coefficients framework. In a nutshell, we are dealing with a particle system of the generic form

$$dX^{i}_{t} = f\Biggl(X^{i}_{t}, \frac{1}{N}\sum_{j=1}^{N}\phi \bigl(X^{j}_{t}\bigr)\Biggr)\,dt + \sigma \bigl(X^{i}_{t}\bigr)\,dW^{i}_{t}, $$

where the Brownian motions $W^{i}$ are independent, and the functions *ϕ*, *f*, and *σ* are such that one gets existence and strong uniqueness of a solution with finite moments, as well as the existence and strong uniqueness of the mean-field limit

$$dX_{t} = f\bigl(X_{t},\mathbb {E}\phi(X_{t})\bigr)\,dt + \sigma(X_{t})\,dW^{1}_{t}. $$

Now, let $(\widetilde{X}^{i}_{t})$ be independent copies of $(X_{t})$ driven by the Brownian motions $W^{i}$. Under strong enough Lipschitz hypotheses on *ϕ*, *f* and *σ*, one typically obtains, for some $C>0$ and all $0\leq t\leq T$,

$$\mathbb {E}\bigl\vert X^{i}_{t}-\widetilde{X}^{i}_{t} \bigr\vert ^{2} \leq C \int_{0}^{t} \mathbb {E}\bigl\vert X^{i}_{\theta}- \widetilde{X}^{i}_{\theta}\bigr\vert ^{2}\,d\theta + C \int_{0}^{t} \mathbb {E}\Biggl\vert \mathbb {E}\phi\bigl(\widetilde{X}^{1}_{\theta}\bigr) - \frac{1}{N}\sum_{j=1}^{N}\phi \bigl(\widetilde{X}^{j}_{\theta}\bigr)\Biggr\vert ^{2}\,d\theta. $$

Using independence arguments one readily gets

$$\mathbb {E}\Biggl\vert \mathbb {E}\phi\bigl(\widetilde{X}^{1}_{\theta}\bigr) - \frac{1}{N}\sum_{j=1}^{N}\phi \bigl(\widetilde{X}^{j}_{\theta}\bigr)\Biggr\vert ^{2} \leq\frac{C}{N}. $$

Using Gronwall’s lemma, one deduces that

$$\mathbb {E}\bigl\vert X^{i}_{t}-\widetilde{X}^{i}_{t} \bigr\vert ^{2} \leq\frac{C}{N}. $$

This method fails when one of *f*, *σ* or *ϕ* is not globally Lipschitz.

In our case the drift *f* is not globally Lipschitz, but of the form (see Eqs. (20), (21), (22))

$$f\Biggl(t,v,\mathbf {q},j,\frac{1}{N}\sum_{i=1}^{N} y^{i}\Biggr) = F_{\alpha}(t,v,\mathbf {q}) - j \bigl(v- \overline{V}^{\alpha\gamma}\bigr) \Biggl(\frac{1}{N}\sum _{i=1}^{N} y^{i}\Biggr). $$

The fact that the first part $F_{\alpha}$ of the drift is only one-sided Lipschitz is easy to overcome. To handle the second part $- j (v- \overline{V}^{\alpha\gamma}) (\frac{1}{N}\sum_{i=1}^{N} y^{i})$ we make use of the following three properties:

- the processes $J_{t}^{\alpha\gamma}$ are positive,
- the processes $y^{i}_{t}$ belong to $[0,1]$,
- in the dynamics of $V_{t}$, the term $- j (v- \overline{V}^{\alpha \gamma}) (\frac{1}{N}\sum_{i=1}^{N} y^{i})$ acts as a mean reverting term, stabilizing the moments of $V_{t}$.

Notice that because in our case the function *f* is not globally Lipschitz, the convergence rate for $\mathbb {E}[ \sup_{0\leq t\leq T} \sum_{\alpha\in \mathcal {P}} \vert R^{i_{\alpha},N}_{t}-{{\widetilde {R}}}^{i_{\alpha}}_{t}\vert ^{2} ]$ is of the order of $1/\sqrt{N}$, as indicated by the inequality (18), instead of $1/N$ in the Lipschitz case.

*Details of our proof*. We now turn to the proof of inequality (18).

By the symmetry of the coupled systems, we can fix the index set $(1_{\alpha}, \alpha\in \mathcal {P})$ and rewrite the SDEs (4) and (9) in the following condensed form: for all $\alpha\in \mathcal {P}$,

19 $$\begin{aligned} R^{1_{\alpha},N}_{t} -{{\widetilde {R}}}^{1_{\alpha}}_{t} ={}& \int_{0}^{t} \bigl( \sigma \bigl(R^{1_{\alpha},N}_{s}\bigr) - \sigma\bigl({{\widetilde {R}}}^{1_{\alpha}}_{s} \bigr) \bigr)\, d {\mathbf {W}}^{1_{\alpha}}_{s} \\ &{}+ \int_{0}^{t} \bigl( B\bigl[s,R^{1_{\alpha},N}_{s}; \mu^{N}_{s}\bigr] - B\bigl[s,{{\widetilde {R}}}^{1_{\alpha}}_{s}; \mathbb{P}_{s}\bigr] \bigr)\,ds, \end{aligned}$$

where we have introduced the empirical measure $\mu^{N}_{\cdot}= \frac{1}{N}\sum_{j=1}^{N} \delta_{R^{j}_{\cdot}}$, the Brownian motion $(\mathbf {W}^{1_{\alpha}}_{t}) = (W^{1_{\alpha},V}_{t}, (B_{t}^{1_{\alpha}\gamma},\gamma\in \mathcal {P}),W^{1_{\alpha},y}_{t},W^{1_{\alpha},x}_{t})$, and the time-marginal law $\mathbb{P}_{s} = \mathbb{P} \circ({{\widetilde {R}}}^{1_{\alpha}}_{s},\alpha\in \mathcal {P})^{-1}$.

We denote by *r* the canonical variable on $\mathbb {R}^{\ell}$, that we decompose in the following set of *p̅* coordinates on $\mathbb {R}^{\overline{p}+6}$:

$$r :=\bigl(r^{\alpha}, \alpha\in \mathcal {P}\bigr) = \bigl(v^{\alpha}, \bigl(j^{\alpha\gamma}; \gamma\in \mathcal {P}\bigr),y^{\alpha},w^{\alpha}, x^{\alpha}; \alpha\in \mathcal {P}\bigr). $$

According to Remark 4.4, the diffusion coefficient is defined as

$$\begin{aligned} \sigma\bigl(r^{\alpha}\bigr)&= \sigma\bigl(v^{\alpha}, \bigl(j^{\alpha\gamma},\gamma\in \mathcal {P}\bigr), y^{\alpha}, w^{\alpha},x^{\alpha}\bigr) \\ &= \left ( \begin{matrix} \sigma^{\alpha}\\ ( \sigma^{J}_{\alpha\gamma}\sqrt{j^{\alpha\gamma}}, \gamma \in \mathcal {P}) \\ \sqrt{ \vert a^{\alpha}_{r} S_{\alpha}(v^{\alpha}) (1-y^{\alpha}) + a^{\alpha}_{d}y^{\alpha} \vert } \chi(y^{\alpha}) \\ 0 \\ \sqrt{ \vert \rho_{x}(v^{\alpha})(1-x^{\alpha}) + \zeta_{x}(v^{\alpha})x^{\alpha} \vert } \chi(x^{\alpha}) \end{matrix} \right ) \end{aligned}$$

and is Lipschitz on the state subspace of the process $(V^{\alpha}_{t},y^{\alpha}_{t}, w^{\alpha}_{t}, x^{\alpha}_{t})$. The drift coefficient is defined as

20 $$\begin{aligned} B\bigl[t,r^{\alpha};\mu\bigr]:= b\bigl(t,r^{\alpha}\bigr) + k\bigl[r^{\alpha};\mu\bigr], \end{aligned}$$

where

21 $$\begin{aligned} b\bigl(t,r^{\alpha}\bigr)&= b\bigl(v^{\alpha}, \bigl(j^{\alpha\gamma},\gamma\in \mathcal {P}\bigr), y^{\alpha}, w^{\alpha},x^{\alpha}\bigr) \\ &= \left ( \begin{matrix} F_{\alpha}(t, v^{\alpha}, \mathbf {q}^{\alpha})\\ ( \theta_{\alpha\gamma} ( \overline{J}^{\alpha\gamma} - j^{\alpha\gamma} ),\gamma\in \mathcal {P})\\ (a^{\alpha}_{r} S_{\alpha}(v^{\alpha}) (1-y^{\alpha}) - a^{\alpha}_{d}y^{\alpha} )\\ c_{\alpha}(v^{\alpha}+ a_{\alpha}- b_{\alpha}w^{\alpha})\\ (\rho_{x}(v^{\alpha})(1-x^{\alpha}) - \zeta_{x}(v^{\alpha})x^{\alpha}) \end{matrix} \right ) \end{aligned}$$

is one-sided Lipschitz in the sense of (10) in Hypothesis 2.1(iii), and *k* is defined as follows. For any probability measure *μ* on $\mathbb {R}^{l}$,

22 $$\begin{aligned} k\bigl[r^{\alpha};\mu\bigr]= \left ( \begin{matrix} - \int_{\mathbb {R}^{\ell}} \sum_{\gamma\in \mathcal {P}} (v^{\alpha}- \overline {V}^{\alpha \gamma}) j^{\alpha\gamma} \frac{1}{c_{\gamma}} {\mathbb {1}}_{\{\eta=\gamma \}} y^{\eta} \mu(d(r^{\eta}; \eta\in \mathcal {P})) \\ 0\\ 0\\ 0\\ 0 \end{matrix} \right ). \end{aligned}$$

#### Remark 4.6

Notice that the characteristic function ${\mathbb {1}}_{\{\eta=\gamma\}}$ is unnecessary in the above definition but, combined with the hypothesis (17), which fixes the constants $\{ c_{\gamma};{\gamma\in \mathcal {P}}\}$, it has the advantage of matching the notations in Eqs. (1) and (4), which helps identifying the interaction kernel in the limit equations. The measure $\mu(d(r^{\eta}; \eta\in \mathcal {P}))$ is on $\mathbb {R}^{\ell}$ whose state coordinates are here labeled in $\mathcal {P}$.

In all the sequel *C* is a positive constant which may change from line to line and is independent of *N* and $0\leq t\leq T$, but it may depend on *T*.

*Step 1. We prove that the processes*$V^{i}_{t}$*have bounded moments of any positive order*. We start with reminding the reader that the CIR processes $(J^{i\gamma})$ have bounded moments of any positive order when their initial conditions enjoy the same property (see e.g. Lemma 2.1 in Alfonsi [11]). In view of the Hypotheses 2.1(i) and (iv), one can show that the same is true for the processes $(V^{i})$ and $(\widetilde{V}^{i})$ by using the following argument. Apply the Itô formula to $(V^{i})^{2q}$, $q\geq1$, till time $\tau_{n} = \inf\{t\geq 0; \vert V^{i}_{t}\vert \geq n\}$, and take expectations to get

$$\begin{aligned} \mathbb {E}\bigl[\bigl(V^{i}_{t\wedge\tau_{n}}\bigr)^{2q}\bigr] ={}& \mathbb {E}\bigl[\bigl(V^{i}_{0}\bigr)^{2q}\bigr] + 2q \int_{0}^{t} \mathbb {E}\bigl[{\mathbb {1}}_{\{s\leq \tau_{n}\}}\bigl(V^{i}_{s}\bigr)^{2q-1} F_{\alpha}\bigl(t, V^{i}_{s} , \mathbf {q}^{i}_{s} \bigr)\bigr]\,ds \\ &{} - 2q \int_{0}^{t}\mathbb {E}\Biggl[{\mathbb {1}}_{\{s\leq \tau_{n}\}}\sum_{ \gamma\in \mathcal {P}} \bigl(V^{i}_{s}\bigr)^{2q-1}\bigl(V^{i}_{s} - \overline{V}^{\alpha \gamma}\bigr) \frac{{J}^{i\gamma}_{s}}{N_{\gamma}} \\ &{}\times \Biggl(\sum_{j=1}^{N} {\mathbb {1}}_{ \{p(j)=\gamma\}}y^{j}_{s} \Biggr) \Biggr]\,ds \\ &{}+ q (2q-1)\int_{0}^{t}\mathbb {E}\bigl[{\mathbb {1}}_{\{s \leq\tau_{n}\}}\bigl(V^{i}_{s} \bigr)^{2q-2} (\sigma_{\alpha})^{2} \bigr]\,ds. \end{aligned}$$

We then observe that

$$- 2q \int_{0}^{t}\mathbb {E}\Biggl[{\mathbb {1}}_{\{s\leq \tau_{n}\}}\sum_{ \gamma\in \mathcal {P}} \bigl(V^{i}_{s}\bigr)^{2q} \frac{{J}^{i\gamma}_{s}}{N_{\gamma}} \Biggl(\sum_{j=1}^{N} {\mathbb {1}}_{\{p(j)= \gamma\}}y^{j}_{s} \Biggr) \Biggr]\,ds $$

is negative and can be ignored. It then remains to use Hypothesis 2.1 and classical arguments to deduce that $\mathbb {E}[(V^{i}_{t})^{n} ]$ is finite for all $n\geq1$.

*Step 2. A first bound for the random variables*$\vert R^{1_{\alpha},N}_{t} -{{\widetilde {R}}}^{1_{\alpha}}_{t}\vert ^{2}$*and*$(\frac{1}{N} \times \sum_{i=1}^{N} (y^{i}_{t} - {\widetilde {y}}^{i}_{t}))^{2}$. Because of the polynomial form of the non-Lipschitz part of the drift, it is not a good idea to introduce the expectation too early in the calculation of the bound for $\vert R^{1_{\alpha},N}_{t} -{{\widetilde {R}}}^{1_{\alpha}}_{t}\vert ^{2}$ or $(\frac{1}{N} \sum_{i=1}^{N} (y^{i}_{t} - {\widetilde {y}}^{i}_{t}))^{2}$, since calculations with expectation lead to terms of the type $\mathbb {E}[(R^{1_{\alpha},N}_{t} -{{\widetilde {R}}}^{1_{\alpha}}_{t})^{2} H]$, where *H* is an unbounded random variable correlated with $R^{1_{\alpha},N}_{t}$. We therefore postpone taking expectations to Step 3.

Apply Itô’s formula to $\vert R^{1_{\alpha},N}_{t} -{{\widetilde {R}}}^{1_{\alpha}}_{t}\vert ^{2}$. We obtain

$$\begin{aligned} \bigl\vert R^{1_{\alpha},N}_{t} -{{\widetilde {R}}}^{1_{\alpha}}_{t} \bigr\vert ^{2} = {}& 2 \int_{0}^{t} \bigl( B\bigl[s,R^{1_{\alpha},N}_{s}; \mu^{N}_{s} \bigr] - B\bigl[s,{{\widetilde {R}}}^{1_{\alpha}}_{s};\mathbb{P}_{s} \bigr] \bigr) \bigl(R^{1_{\alpha},N}_{s} -{{\widetilde {R}}}^{1_{\alpha}}_{s} \bigr)\,ds \\ & {}+ \int_{0}^{t} \bigl\vert \sigma \bigl(R^{1_{\alpha},N}_{s}\bigr) - \sigma\bigl({{\widetilde {R}}}^{1_{\alpha}}_{s} \bigr)\bigr\vert ^{2}\,ds + M^{1_{\alpha},N}_{t}, \end{aligned}$$

where

$$M^{1_{\alpha},N}_{t}=2\int_{0}^{t} \bigl(R^{1_{\alpha},N}_{s} -{{\widetilde {R}}}^{1_{\alpha}}_{s}\bigr) \bigl( \sigma\bigl(R^{1_{\alpha},N}_{s}\bigr) - \sigma\bigl({ {\widetilde {R}}}^{1_{\alpha}}_{s}\bigr) \bigr) \,d {\mathbf {W}}^{1_{\alpha}}_{s} $$

is a martingale. By Itô isometry and the result in Step 1 above, $\sup_{0\leq t\leq T}\mathbb {E}\vert M^{1_{\alpha},N}_{t}\vert ^{2} \leq C$. Applying the Lipschitz and one-sided-Lipschitz properties for *σ* and *b*, we obtain

23 $$\begin{aligned} \begin{aligned}[b] \bigl\vert R^{1_{\alpha},N}_{t} -{{\widetilde {R}}}^{1_{\alpha}}_{t}\bigr\vert ^{2} \leq{}& 2 \int _{0}^{t} \bigl( k\bigl[s,R^{1_{\alpha},N}_{s}; \mu^{N}_{s}\bigr] - k\bigl[s,{{\widetilde {R}}}^{1_{\alpha}}_{s}; \mathbb{P}_{s}\bigr] \bigr) \bigl(R^{1_{\alpha},N}_{s} -{ {\widetilde {R}}}^{1_{\alpha}}_{s}\bigr)\,ds \\ &{} + C \int_{0}^{t} \bigl\vert R^{1_{\alpha},N}_{s} -{{\widetilde {R}}}^{1_{\alpha}}_{s}\bigr\vert ^{2}\,ds + M^{1_{\alpha},N}_{t}. \end{aligned} \end{aligned}$$

Now, we are interested in $( k[s,R^{1_{\alpha},N}_{s}; \mu^{N}_{s}] - k[s,{{\widetilde {R}}}^{1_{\alpha}}_{s};\mathbb{P}_{s}] ) (R^{1_{\alpha},N}_{s} -{{\widetilde {R}}}^{1_{\alpha}}_{s}) $. We introduce the empirical measure of the coupling system $\widetilde{\mu}^{N}_{\cdot}= \frac{1}{N}\sum_{i=1}^{N} \delta_{{{\widetilde {R}}}^{i}_{\cdot}}$ and write

24 $$\begin{aligned} \bigl( k\bigl[R^{1_{\alpha},N}_{s}; \mu^{N}_{s}\bigr] - k\bigl[{{\widetilde {R}}}^{1_{\alpha}}_{s}; \mathbb {P}_{s}\bigr] \bigr)={}& \bigl( k\bigl[R^{1_{\alpha},N}_{s}; \mu^{N}_{s}\bigr] - k\bigl[{{\widetilde {R}}}^{1_{\alpha}}_{s}; \widetilde{\mu}^{N}_{s}\bigr] \bigr) \\ &{} + \bigl( k\bigl[{{\widetilde {R}}}^{1_{\alpha}}_{s}; \widetilde{ \mu}^{N}_{s}\bigr] - k\bigl[{{\widetilde {R}}}^{1_{\alpha}}_{s}; \mathbb{P}_{s}\bigr] \bigr). \end{aligned}$$

We consider in turn the two terms in the right-hand side of (24).

First, from the definition of *k* in (22) we get

$$\begin{aligned} & \bigl( k\bigl[R^{1_{\alpha},N}_{s}; \mu^{N}_{s} \bigr] - k\bigl[{{\widetilde {R}}}^{1_{\alpha}}_{s};\widetilde { \mu}^{N}_{s}\bigr] \bigr) \bigl(R^{1_{\alpha},N}_{s} -{{\widetilde {R}}}^{1_{\alpha}}_{s}\bigr) \\ &\quad = \Biggl( \frac{1}{N} \sum_{j=1}^{N} \sum_{\gamma\in \mathcal {P}} \biggl[ -\bigl(V^{1_{\alpha}}_{s} - \overline{V}^{\alpha\gamma}\bigr) J^{1_{\alpha }\gamma}_{s} \frac{1}{c_{\gamma}} {\mathbb {1}}_{\{p(j) = \gamma\}} y^{j}_{s} \\ &\qquad {}+ \bigl(\widetilde{V}^{1_{\alpha}}_{s} - \overline{V}^{\alpha\gamma}\bigr) {J}^{1_{\alpha}\gamma}_{s} \frac{1}{c_{\gamma}} {\mathbb {1}}_{\{p(j) = \gamma\}} \widetilde{y}^{j}_{s} \biggr] \Biggr) \bigl(V^{1_{\alpha}}_{s} - {\widetilde {V}}^{1_{\alpha}}_{s} \bigr) \\ &\quad = - \bigl(V^{1_{\alpha}}_{s} - {\widetilde {V}}^{1_{\alpha}}_{s} \bigr)^{2} \Biggl(\sum_{\gamma\in \mathcal {P}} J^{1_{\alpha}\gamma}_{s} \frac{1}{N} \sum _{j=1}^{N} \frac{1}{c_{\gamma}} {\mathbb {1}}_{\{p(j) = \gamma\}} y^{j}_{s} \Biggr) \\ & \qquad {}+ \bigl(V^{1_{\alpha}}_{s} - {\widetilde {V}}^{1_{\alpha}}_{s} \bigr) \Biggl( \sum_{\gamma\in \mathcal {P}} {J}^{1_{\alpha}\gamma}_{s} \bigl(\widetilde{V}^{1_{\alpha}}_{s} - \overline {V}^{\alpha\gamma} \bigr) \frac{1}{N} \sum_{j=1}^{N} \frac{1}{c_{\gamma}} {\mathbb {1}}_{\{p(j) = \gamma\}} \bigl(\widetilde{y}^{j}_{s} - y^{j}_{s}\bigr) \Biggr). \end{aligned}$$

Since the $(J^{1_{\alpha}\gamma}_{t})$ and the $(y^{i}_{t},i=1,\ldots,N)$ are positive, the first term in the right-hand side is negative. We bound the second term by using Young’s inequality:

25 $$\begin{aligned} & \bigl(k\bigl[R^{1_{\alpha},N}_{s}; \mu^{N}_{s}\bigr] - k\bigl[{{\widetilde {R}}}^{1_{\alpha}}_{s}; \widetilde{\mu}^{N}_{s}\bigr] \bigr) \bigl(R^{1_{\alpha},N}_{s} -{{\widetilde {R}}}^{1_{\alpha}}_{s}\bigr) \\ & \quad \leq\frac{1}{2} \bigl(V^{1_{\alpha}}_{s} - {\widetilde {V}}^{1_{\alpha}}_{s}\bigr)^{2} \\ &\qquad {}+ \frac{1}{2} \Biggl( \sum_{\gamma\in \mathcal {P}} {J}^{1_{\alpha}\gamma}_{s} \bigl(\widetilde{V}^{1_{\alpha}}_{s} - \overline{V}^{\alpha\gamma}\bigr) \frac{1}{N} \sum _{j=1}^{N} \frac{1}{c_{\gamma}} {\mathbb {1}}_{\{p(j) = \gamma\}} \bigl(\widetilde{y}^{j}_{s} - y^{j}_{s}\bigr) \Biggr)^{2}. \end{aligned}$$

Next we consider the second contribution coming from the right-hand side of (24). By Young’s inequality

26 $$\begin{aligned} \bigl(k\bigl[{{\widetilde {R}}}^{1_{\alpha}}_{s};\widetilde{ \mu}^{N}_{s}\bigr] - k\bigl[{{\widetilde {R}}}^{1_{\alpha}}_{s}; \mathbb{P}_{s}\bigr] \bigr) \bigl(R^{1_{\alpha},N}_{s} -{ {\widetilde {R}}}^{1_{\alpha}}_{s}\bigr) \leq\frac{1}{2} \bigl\vert R^{1_{\alpha},N}_{s} -{{\widetilde {R}}}^{1_{\alpha}}_{s}\bigr\vert ^{2} + \frac{1}{2} \bigl(\zeta^{1_{\alpha}}_{s} \bigr)^{2}, \end{aligned}$$

where

$$\zeta^{1_{\alpha}}_{s}:= k\bigl[{{\widetilde {R}}}^{1_{\alpha}}_{s}; \widetilde{\mu}^{N}_{s}\bigr] - k\bigl[{ {\widetilde {R}}}^{1_{\alpha}}_{s};\mathbb{P}_{s}\bigr] $$

is such that $\sup_{0\leq t\leq T}\mathbb {E}\vert \zeta^{1_{\alpha}}_{s}\vert ^{2} \leq \frac {C}{N}$. Indeed, as the $({{\widetilde {R}}}^{i})$ are i.i.d. with law $\mathbb{P}$, $k[{{\widetilde {R}}}^{1_{\alpha}}_{s};\mathbb{P}_{s}]$ is the conditional expectation

$$\begin{aligned} k\bigl[{{\widetilde {R}}}^{1_{\alpha}}_{s};\mathbb{P}_{s}\bigr] & = \mathbb {E}\bigl[k\bigl({{\widetilde {R}}}^{1_{\alpha}}_{s},{{\widetilde {R}}}^{j}_{s} \bigr) / \sigma\bigl( {{\widetilde {R}}}^{1_{\alpha}}_{u}; u\leq s\bigr) \bigr] \end{aligned}$$

for any $j \neq1_{\alpha}$, where we have set $k(r^{\alpha},r^{\eta})= \sum_{\gamma\in \mathcal {P}} (v^{\alpha}- \overline{V}^{\alpha\gamma}) j^{\alpha\gamma} \frac{1}{c_{\gamma}} {\mathbb {1}}_{\{\eta= \gamma\}} y^{\eta}$, and the symbol *σ* stands for sigma algebra (which must not be confused with the above diffusion coefficient). Thus

$$\begin{aligned} \mathbb {E}\bigl\vert \zeta^{1_{\alpha}}_{s} \bigr\vert ^{2} ={}& \mathbb {E}\Biggl\vert \frac{1}{N} \sum_{j= 1}^{N} k\bigl({{\widetilde {R}}}^{1_{\alpha}}_{s}, {{\widetilde {R}}}^{j}_{s} \bigr) - \mathbb {E}\bigl[k\bigl({{\widetilde {R}}}^{1_{\alpha}}_{s},{ {\widetilde {R}}}^{j}_{s}\bigr) / \sigma\bigl( {{\widetilde {R}}}^{1_{\alpha}}_{u}; u\leq s\bigr) \bigr] \Biggr\vert ^{2} \\ \leq{}&2 \mathbb {E}\biggl\vert \frac{1}{N} \sum_{j \neq1_{\alpha}} k\bigl({{\widetilde {R}}}^{1_{\alpha}}_{s}, {{\widetilde {R}}}^{j}_{s} \bigr) - \mathbb {E}\bigl[k\bigl({{\widetilde {R}}}^{1_{\alpha}}_{s},{{\widetilde {R}}}^{j}_{s}\bigr) / \sigma\bigl( {{\widetilde {R}}}^{1_{\alpha}}_{u}; u\leq s\bigr) \bigr] \biggr\vert ^{2} \\ &{} + \frac{2}{N}\mathbb {E}\Bigl\vert k\bigl({{\widetilde {R}}}^{1_{\alpha}}_{s},{ {\widetilde {R}}}^{1_{\alpha}}_{s}\bigr) - \mathbb {E}\bigl[k\bigl(r^{\alpha},{ {\widetilde {R}}}^{1_{\alpha}}_{s}\bigr)\bigr]\big| _{\{r^{\alpha}= {{\widetilde {R}}}^{1_{\alpha}}_{s}\}} \Bigr\vert ^{2}\\ \leq{}&\frac{C}{N}. \end{aligned}$$

Combining (23) with the last inequalities (25), (24), and (26),

$$\begin{aligned} \bigl\vert R^{1_{\alpha},N}_{t} -{{\widetilde {R}}}^{1_{\alpha}}_{t} \bigr\vert ^{2} \leq{}& C\int_{0}^{t} \bigl\vert R^{1_{\alpha},N}_{s} -{{\widetilde {R}}}^{1_{\alpha}}_{s} \bigr\vert ^{2}\,ds \\ &{} + C\int_{0}^{t} \sum _{\gamma\in \mathcal {P}} \bigl(J^{1_{\alpha}\gamma}_{s} \bigr)^{2} \bigl(\widetilde{V}^{1_{\alpha}}_{s} - \overline{V}^{\alpha\gamma}\bigr)^{2} \Biggl(\frac{1}{N} \sum _{j=1}^{N} \bigl(\widetilde{y}^{j}_{s} - y^{j}_{s}\bigr) \Biggr)^{2}\,ds \\ &{} + M^{1_{\alpha},N}_{t} + \frac{1}{2} \bigl( \zeta^{1_{\alpha}}_{t}\bigr)^{2}. \end{aligned}$$

By Gronwall’s lemma and integration by parts, we have

27 $$ \begin{aligned}[b] &\bigl\vert R^{1_{\alpha},N}_{t} -{{\widetilde {R}}}^{1_{\alpha}}_{t}\bigr\vert ^{2} \\ &\quad \leq C\int_{0}^{t} \sum _{\gamma\in \mathcal {P}} \bigl(J^{1_{\alpha}\gamma}_{s} \bigr)^{2} \bigl(\widetilde{V}^{1_{\alpha}}_{s} - \overline{V}^{\alpha\gamma}\bigr)^{2} \Biggl(\frac{1}{N} \sum _{j=1}^{N} \bigl(\widetilde{y}^{j}_{s} - y^{j}_{s}\bigr) \Biggr)^{2}\,ds + Z^{1_{\alpha}}_{t}, \end{aligned} $$

where for all $t\in[0,T]$, since $(M^{1_{\alpha},N}_{t})$ is a martingale,

$$\begin{aligned} \mathbb {E}\bigl[Z^{1_{\alpha}}_{t}\bigr]& = \mathbb {E}\biggl[M^{1_{\alpha},N}_{t} + \frac{1}{2} \bigl(\zeta ^{1_{\alpha}}_{t} \bigr)^{2}+\int_{0}^{t} C\exp\bigl(C(t-s)\bigr) \biggl(M^{1_{\alpha},N}_{s} + \frac {1}{2} \bigl(\zeta^{1_{\alpha}}_{s}\bigr)^{2} \biggr)\,ds \biggr] \\ &\leq C \sup_{0\leq t\leq T}\mathbb {E}\bigl\vert \zeta^{1_{\alpha}}_{s} \bigr\vert ^{2} \leq\frac{C}{N}. \end{aligned} $$

We now set

$$\overline {\delta y}_{t} = \frac{1}{N} \sum_{i=1}^{N} \bigl(y^{i}_{t} - {\widetilde {y}}^{i}_{t}\bigr). $$

Defining the drift and diffusion for processes $y^{i}$ by

$$\begin{aligned} b^{\alpha}_{y}(y,v) &=a^{\alpha}_{r} S_{\alpha}(v) (1-y) - a^{\alpha}_{d} y, \\ \sigma^{\alpha}_{y}(y,v) &= \sqrt{ \bigl\vert a^{\alpha}_{r} S_{\alpha}(v) (1-y) + a^{\alpha}_{d} y\bigr\vert } \chi(y), \end{aligned}$$

we have

$$\begin{aligned} (\overline {\delta y}_{t})^{2} = {}& \Biggl( \int_{0}^{t} \frac{1}{N}\sum_{i=1}^{N} \bigl(b^{\alpha}_{y}\bigl(y_{s}^{i},V_{s}^{i} \bigr) - b^{\alpha}_{y}\bigl({\widetilde {y}}_{s}^{i}, {\widetilde {V}}_{s}^{i}\bigr) \bigr)\,ds \\ &{}+ \int_{0}^{t} \frac {1}{N}\sum _{i=1}^{N} \bigl(\sigma^{\alpha}_{y} \bigl(y_{s}^{i},V_{s}^{i}\bigr) - \sigma^{\alpha}_{y}\bigl({\widetilde {y}}_{s}^{i}, {\widetilde {V}}_{s}^{i}\bigr) \bigr)\,dW_{s}^{i} \Biggr)^{2} \\ \leq{}& 2 \Biggl( \int_{0}^{t} \frac{1}{N}\sum_{i=1}^{N} \bigl(b^{\alpha}_{y}\bigl(y_{s}^{i},V_{s}^{i} \bigr) - b^{\alpha}_{y}\bigl({\widetilde {y}}_{s}^{i}, {\widetilde {V}}_{s}^{i}\bigr) \bigr)\,ds \Biggr)^{2} \\ &{}+ 2 \Biggl( \int_{0}^{t} \frac{1}{N} \sum_{i=1}^{N} \bigl(\sigma^{\alpha}_{y} \bigl(y_{s}^{i},V_{s}^{i}\bigr) - \sigma ^{\alpha}_{y}\bigl({\widetilde {y}}_{s}^{i}, {\widetilde {V}}_{s}^{i}\bigr) \bigr)\,dW_{s}^{i} \Biggr)^{2}. \end{aligned}$$

Notice that the processes $(\overline{Z}_{t})$, defined by

$$\overline{Z}_{t} := 2 \Biggl( \int_{0}^{t} \frac{1}{N}\sum_{i=1}^{N} \bigl( \sigma ^{\alpha}_{y}\bigl(y_{s}^{i},V_{s}^{i} \bigr) - \sigma^{\alpha}_{y}\bigl({\widetilde {y}}_{s}^{i}, {\widetilde {V}}_{s}^{i}\bigr) \bigr)\,dW_{s}^{i} \Biggr)^{2} $$

is such that $\sup_{0\leq t\leq T}\mathbb {E}(\overline{Z}_{t})^{2} \leq\frac{C}{N}$. Since $b^{\alpha}_{y}$ and $\sigma^{\alpha}_{y}$ are Lipschitz on $[0,1]\times \mathbb {R}$, we get

$$\begin{aligned} (\overline {\delta y}_{t})^{2} &\leq \int_{0}^{t} C ( \overline {\delta y}_{s})^{2}\,ds + \int_{0}^{t} C \Biggl(\frac {1}{N}\sum_{i=1}^{N} \bigl\vert V^{i}_{s} - {\widetilde {V}}^{i}_{s} \bigr\vert \Biggr)^{2}\,ds +\overline{Z}_{t} \\ &\leq \int_{0}^{t} C (\overline {\delta y}_{s})^{2} \,ds + \int_{0}^{t} \frac{C}{N}\sum _{i=1}^{N} \bigl\vert R^{i,N}_{s} - {\widetilde {R}}^{i}_{s}\bigr\vert ^{2}\,ds + \overline{Z}_{t}. \end{aligned}$$

Combining again Gronwall’s lemma and integration by parts we obtain

28 $$\begin{aligned} \overline {\delta y}_{t}^{2} \leq{}& \int_{0}^{t} \frac{C}{N}\sum_{i=1}^{N} \bigl\vert R^{i,N}_{s} - {\widetilde {R}}^{i}_{s}\bigr\vert ^{2}\,ds \\ &{} + \int_{0}^{t} C e^{C(t-s)} \Biggl( \int_{0}^{s} \frac{C}{N}\sum_{i=1}^{N} \bigl\vert R^{i,N}_{\theta}- {\widetilde {R}}^{i}_{\theta}\bigr\vert ^{2}\,d\theta \Biggr)\,ds \\ &{}+\overline {Z}_{t} + \int_{0}^{t} C e^{C(t-s)} \overline{Z}_{s} \,ds \\ \leq{}& C \int_{0}^{t} \frac{1}{N}\sum _{i=1}^{N} \bigl\vert R^{i,N}_{s} - {\widetilde {R}}^{i}_{s}\bigr\vert ^{2}\,ds + \overline{Z}_{t} + \int_{0}^{t} C e^{C(t-s)} \overline{Z}_{s} \,ds. \end{aligned}$$

*Step 3. The bound for*$\mathbb {E}[ \sup_{0\leq t\leq T} \sum_{\alpha\in \mathcal {P}} \vert R^{i_{\alpha},N}_{t}-{{\widetilde {R}}}^{i_{\alpha}}_{t}\vert ^{2} ]$. Combining the last inequality (28) with (27), we have

$$\begin{aligned} \overline {\delta y}_{t}^{2} \leq{}& C \int_{0}^{t} \int_{0}^{s} \Biggl(\frac{C}{N}\sum _{i=1}^{N} \sum _{\gamma\in \mathcal {P}} \bigl(J^{i\gamma}_{\theta}\bigr)^{2} \bigl(\widetilde{V}^{i}_{\theta}- \overline{V}^{\alpha\gamma}\bigr)^{2} \Biggr) (\overline {\delta y}_{\theta})^{2} \,d\theta \,ds \\ &{} +\int_{0}^{t} \frac{C}{N}\sum _{i=1}^{N} Z^{i}_{s} \,ds + \overline{Z}_{t} + \int_{0}^{t} C e^{C(t-s)} \overline{Z}_{s} \,ds \\ ={}& C \int_{0}^{t} (t-s) \Biggl( \frac{C}{N}\sum_{i=1}^{N} \sum _{\gamma\in \mathcal {P}} \bigl(J^{i\gamma}_{s} \bigr)^{2} \bigl(\widetilde{V}^{i}_{s} - \overline{V}^{\alpha\gamma}\bigr)^{2} \Biggr) (\overline {\delta y}_{s})^{2} \,ds \\ &{} +\int_{0}^{t} \frac{C}{N}\sum _{i=1}^{N} Z^{i}_{s} \,ds + \overline{Z}_{t} + \int_{0}^{t} C e^{C(t-s)} \overline{Z}_{s} \,ds \\ ={}& C \int_{0}^{t} (t-s)\mathbb {E}\biggl[ \sum _{\gamma\in \mathcal {P}} \bigl(J^{1\gamma}_{s} \bigr)^{2} \bigl(\widetilde{V}^{1}_{s} - \overline{V}^{\alpha\gamma}\bigr)^{2} \biggr] (\overline {\delta y}_{s})^{2} \,ds + \gamma_{t} \\ &{} +\int_{0}^{t} \frac{C}{N}\sum _{i=1}^{N} Z^{i}_{s} \,ds + \overline {Z}_{t} + \int_{0}^{t} C e^{C(t-s)} \overline{Z}_{s} \,ds, \end{aligned}$$

where

$$\begin{aligned} \gamma_{t} := {}&C \int_{0}^{t} (t-s) \Biggl\{ \Biggl(\frac{C}{N}\sum_{i=1}^{N} \sum_{\gamma\in \mathcal {P}} \bigl(J^{i\gamma}_{s} \bigr)^{2} \bigl(\widetilde{V}^{i}_{s} - \overline{V}^{\alpha\gamma}\bigr)^{2}\Biggr) \\ &{} - \mathbb {E}\biggl[ \sum_{\gamma\in \mathcal {P}} \bigl(J^{1\gamma}_{s} \bigr)^{2} \bigl(\widetilde{V}^{1}_{s} - \overline{V}^{\alpha\gamma}\bigr)^{2}\biggr] \Biggr\} (\overline {\delta y}_{s})^{2}\,ds \end{aligned} $$

is such that $\sup_{0\leq t\leq T}\mathbb {E}(\gamma_{t})^{2} \leq\frac{C}{N}$, since $(\overline {\delta y}_{s})^{2}\leq1$ a.s. Taking the expectation of the last inequality, we get

$$\begin{aligned} \mathbb {E}\bigl[\overline {\delta y}_{t}^{2}\bigr] & \leq C \int _{0}^{t} (t-s)\mathbb {E}\biggl[ \sum _{\gamma\in \mathcal {P}} \bigl(J^{1\gamma}_{s} \bigr)^{2} \bigl(\widetilde{V}^{1}_{s} - \overline{V}^{\alpha\gamma}\bigr)^{2} \biggr] \mathbb {E}[\overline {\delta y}_{s}]^{2}\,ds + \frac{C}{\sqrt{N}} \\ &\leq\frac{C}{\sqrt{N}} \end{aligned}$$

by applying again Gronwall’s lemma in the case of a non-decreasing remainder. Coming back to (27), we get

$$\begin{aligned} \mathbb {E}\Biggl[\frac{1}{N}\sum_{i=1}^{N} \bigl\vert R^{i,N}_{t} - {\widetilde {R}}^{i}_{t} \bigr\vert ^{2} \Biggr] \leq{}& C \int_{0}^{t} \mathbb {E}\biggl[\sum_{\gamma\in \mathcal {P}} \bigl(J^{1\gamma}_{s} \bigr)^{2} \bigl(\widetilde{V}^{1}_{s} - \overline{V}^{\alpha\gamma}\bigr)^{2}\biggr] \mathbb {E}\bigl[( \overline {\delta y}_{s})^{2}\bigr]\,ds \\ &{} + \frac{1}{N}\sum_{i=1}^{N} \mathbb {E}\bigl[Z^{i}_{t}\bigr] + \mathbb {E}[\beta_{t}], \end{aligned}$$

where again

$$\begin{aligned} \beta_{t} :={}&C \int_{0}^{t} \Biggl\{ \Biggl(\frac{1}{N}\sum_{i=1}^{N} \sum_{\gamma \in \mathcal {P}} \bigl(J^{i\gamma}_{s} \bigr)^{2} \bigl(\widetilde{V}^{i}_{s} - \overline{V}^{\alpha\gamma}\bigr)^{2}\Biggr) \\ &{} - \mathbb {E}\biggl[ \sum_{\gamma\in \mathcal {P}} \bigl(J^{1\gamma}_{s} \bigr)^{2} \bigl(\widetilde{V}^{1}_{s} - \overline{V}^{\alpha\gamma}\bigr)^{2}\biggr] \Biggr\} (\overline {\delta y}_{s})^{2}\,ds, \end{aligned} $$

is such that $\sup_{0\leq t\leq T}\mathbb {E}(\beta_{t})^{2} \leq\frac{C}{N}$. Using (17), this ends the proof of the proposition.  □

## Conclusion

In this note we have set the work published in [1] on a totally rigorous footing. In doing so we also have shed some new light on the way to incorporate noise in the ion channels equations for the Hodgkin–Huxley model and in the amount of neurotransmitters at the synapses in both the Hodgkin–Huxley and the FitzHugh–Nagumo models.

The techniques in this paper could be extended to a more generic form of interaction kernel $k[r;\mu]$ in (22). Notice also that the Hypothesis 2.1(iii) should allow one to prove the convergence in time to equilibrium of the mean-field limits.

## Footnotes

1 Similarly, Counterexample 1 in [4] contradicts the results on neuronal models claimed at the end of Sect. 1 and in Appendix B of [5].
